# Enhancing early-stage breakthrough technology identification: A CatBoost model optimized with the SABO algorithm

**DOI:** 10.1371/journal.pone.0356903

**Published:** 2026-09-15

**Authors:** Ming Lei, Yin Yu

**Affiliations:** School of Management, Beijing Institute of Technology, Beijing, China; USTC: University of Science and Technology of China, CHINA

## Abstract

Early identification of breakthrough technologies is essential for strategic R&D planning, but it remains a major challenge in technology analysis and strategic management. In their early stages, these technologies often appear in patent data with weak and ambiguous indicators and are greatly outnumbered by incremental innovations. This results in low feature discriminability and severe class imbalance. To address these challenges, we present a new automated framework that combines CatBoost with the SABO heuristic optimization algorithm. In this approach, CatBoost’s key hyperparameters are treated as SABO search agents and are optimized to improve the minority-class F1-score using stratified cross-validation. This approach directs the model to achieve high sensitivity and robustness, given the imbalanced data and subtle indicators. Experimental validation using the Derwent Innovation patent data set shows that SABO-tuned CatBoost significantly outperforms both standard CatBoost and other optimizer-based models in F1-score for breakthrough identification. Model interpretation through feature importance and SHAP analysis identifies discriminative indicators for early-stage breakthroughs. The proposed framework offers an effective and interpretable tool for technology forecasting under these constraints, supporting early opportunity discovery and data-driven innovation strategy.

## Introduction

Early identification of breakthrough technologies remains a fundamental and ongoing challenge in science and technology policy and innovation management. Accurately distinguishing significant, trajectory-changing advances from incremental progress is crucial because it directly informs strategic research funding, corporate R&D portfolios, and national competitive positioning [1]. However, this task is inherently difficult due to the uncertainty inherent in innovation and the large volume of modern scientific output [2].

The scientific consensus holds that the status of breakthrough research is ultimately adjudicated by the scholarly community [3]. For decades, this judgment has relied on expert peer review, which is valued for its depth of insight but is increasingly viewed as limited by subjectivity, cognitive biases, and poor scalability as scientific literature grows rapidly. Because of the surge in publications, relying solely on expert assessment to quickly identify seminal works is now impractical [4].

This challenge led to the adoption of bibliometric analysis, with forward citation counts becoming the main proxy for research impact. Schoenmakers et al., for example, used semiconductor patent data to show that highly cited patents often indicate breakthrough innovations [5]. Another line of research shifted focus from citation volume to citation structure, based on the hypothesis that transformative research changes the knowledge architecture of its field [6]. Min et al. supported this perspective by modeling knowledge networks using citation linkages generated by a publication [7]. Similarly, Dahlin, et al. linked breakthrough technologies to divergence in patent citation patterns, suggesting that greater structural disparity correlates with higher innovativeness [8]. Despite their theoretical appeal, these citation-based methods have a critical drawback: the time required for citations to accumulate introduces a substantial lag, often years, between publication and identification. This delay reduces their usefulness for real-time decision-making in rapidly changing technological environments.

To address this temporal constraint, researchers have used machine learning (ML) techniques that rely on intrinsic features available at the time of publication. Structured metadata, such as the number of claims, backward citations, and inventor counts, has shown promise in predicting patent value [9]. A significant development is the application of deep learning to textual content. For example, Park and Sohn showed that hybrid models combining CNNs and Bi-LSTMs can extract detailed semantic and sequential patterns from abstracts and claims, achieving high precision in grading patents and highlighting the importance of contextual analysis beyond conventional metrics [10].

Nevertheless, these approaches face two main challenges: the weak and underdeveloped signals typical of early-stage breakthroughs, and the significant class imbalance, as seminal works represent only a small minority. Current predictive models are often ill-equipped for this high-stakes, low-signal context, as they may fail to detect subtle structural cues or lack the robustness needed to handle severe class imbalance.

To address this specific bottleneck, this paper presents a ML framework that combines advanced hyperparameter optimization with rigorous feature selection. Drawing on the predictive value of structural metrics, and given the ability of ML to process these complex feature sets, our approach introduces a key refinement: the use of the Subtraction-Average-Based Optimizer (SABO) to fine-tune a CatBoost classifier [11,12]. This process increases the model’s sensitivity to subtle structural signals and improves its performance with imbalanced classes. By tailoring the model to the challenges of early identification, our framework provides a more robust, reliable, and timely tool for identifying groundbreaking research at its inception, integrating leading approaches in the field into a practical and scalable solution.

## Literature review

In recent years, both scientists and research funding organizations have recognized the importance of breakthrough research and have examined ways to support it [13]. However, breakthrough research often requires time to gain broad recognition, which makes early identification difficult [14]. This type of research is mainly published in scientific papers, and those with groundbreaking content are called “breakthrough papers.” As a result, identifying these papers has become a central focus in academia.

The scientific community generally agrees that it determines whether a scientific work qualifies as breakthrough research [3]. Peer review, a traditional method for identifying breakthrough papers, is considered effective. However, this approach is often criticized for subjectivity and potential bias. Moreover, the rapid increase in the volume of scientific publications has made relying solely on expert knowledge to quickly identify breakthrough papers inefficient [4].

Bibliometrics offers an alternative method for identifying breakthrough papers by using quantitative indicators such as citation counts, citation patterns, and interdisciplinarity. This approach reduces the subjectivity linked to peer review. Citation counts, among the earliest and most widely used metrics, are often used to identify highly cited papers, which are commonly considered proxies for scientific breakthroughs [15]. Beyond citation frequency, other indicators, such as geographical diversity and interdisciplinary reach, have also been examined for their potential to indicate groundbreaking research. Further studies indicate that detailed analysis of citation patterns can reveal the disruptive potential of publications. For example, Funk and Owen-Smith introduced a network-based metric to systematically assess how much a publication disrupts its knowledge base by examining whether subsequent studies cite the focal paper while omitting its foundational references [16]. This approach shifts the focus from citation volume to intellectual influence on later research. Building on this method, Wu, et al. applied the disruptiveness metric to analyze the rapid increase in China’s scientific output [17]. Their study showed a clear contrast: although China experienced significant growth in publication numbers, the average disruptiveness of its research remained lower than that of established scientific leaders, such as the United States. This finding indicated that, during the period studied, China’s scientific system was more effective at consolidating and extending existing research paradigms than at initiating transformative new directions. More recently, scholars such as Min et al. have used citation structure analysis to track changes over time in how individual papers are cited, providing a dynamic approach for predicting breakthrough potential [7].

Citations accumulate over time, resulting in a time lag that limits the effectiveness of citation analysis for early identification of breakthrough innovations. ML can use features from historical breakthrough patents to predict the breakthrough potential of newly published patents. This method links a patent’s early-stage characteristics to its eventual breakthrough status, allowing identification based on initial features rather than later citation counts. ML methods include supervised, semi-supervised, and unsupervised learning, with supervised learning being especially common in prediction tasks [18]. Instead of depending on citation data that develop slowly, these approaches aim to identify breakthrough research using features available at publication, such as textual content, author prominence, and journal influence.

A growing body of research shows the feasibility of this paradigm, with most studies focusing on predicting citation counts using ML. Yan et al. were the first to use content, author, and venue features with models such as Linear Regression and Support Vector Regression [19]. Singh et al. confirmed the high value of these features for citation prediction using SVM and SVR models [20]. Later, Chen and Zhang introduced a more refined set of content and author features, achieving better performance with ensemble methods such as Random Forest and Gradient Boosted Regression Trees [21]. More recently, Abrishami and Aliakbary improved the methodology by using a sequence-to-sequence model based on RNNs to capture complex temporal dynamics in citation sequences [22].

Beyond citation prediction, researchers have developed ML frameworks to identify breakthrough research more directly. Wang et al. proposed a dual “others-self” evaluation approach that integrates peer citations with author claims and automated the identification process using models such as SVM and BERT [23]. In patent analysis, Park and Sohn developed a deep learning framework that combines textual analysis of abstracts and claims with traditional patent indices [10]. Using a multi-source data perspective, Li et al. introduced a framework for early identification of potential breakthrough research by integrating scientific publications with Twitter data, using solar cell technology as a case study [24].

However, a recognized challenge in this domain is the inherent trade-off between prediction accuracy and early intervention. Models that use less post-publication data generally have more difficulty achieving high predictive performance because they lose the strong signal provided by early citation trends.

Building on these foundations, this paper aims to identify potential breakthrough technologies using ML models. First, we extract from patents a set of predictive indicators that act as early signals of breakthrough potential. Next, we train several models on historical data of recognized breakthrough patents to select the optimal base predictor. To address the limited accuracy of current ML methods in early identification, we propose an enhanced approach that uses the Subtraction-Average-Based Optimizer (SABO) to fine-tune a CatBoost classifier.

Hyperparameter optimization is critical for maximizing the performance of machine learning models, particularly when dealing with imbalanced data. Hybrid optimization approaches combining genetic algorithms and particle swarm optimization have demonstrated effectiveness for SVM parameter tuning in fault diagnosis tasks with limited data [25]. While such hybrid approaches are powerful, the SABO algorithm used in this study offers a distinct advantage through its population-based update mechanism that distributes update influences across all agents, reducing the risk of premature convergence. The addition of Lévy-flight step sizes further enhances global exploration capability, making it particularly suitable for navigating the complex hyperparameter landscape of gradient boosting models.

Network-based AUC optimization methods have proven effective for imbalanced classification tasks by maximizing the area under the ROC curve while accounting for feature interactions [24]. In our study, the use of F1-score as the optimization objective serves a similar purpose—balancing precision and recall to identify the minority class (breakthroughs) without being overwhelmed by the majority class.

## Materials and methods

**Fig 1:**
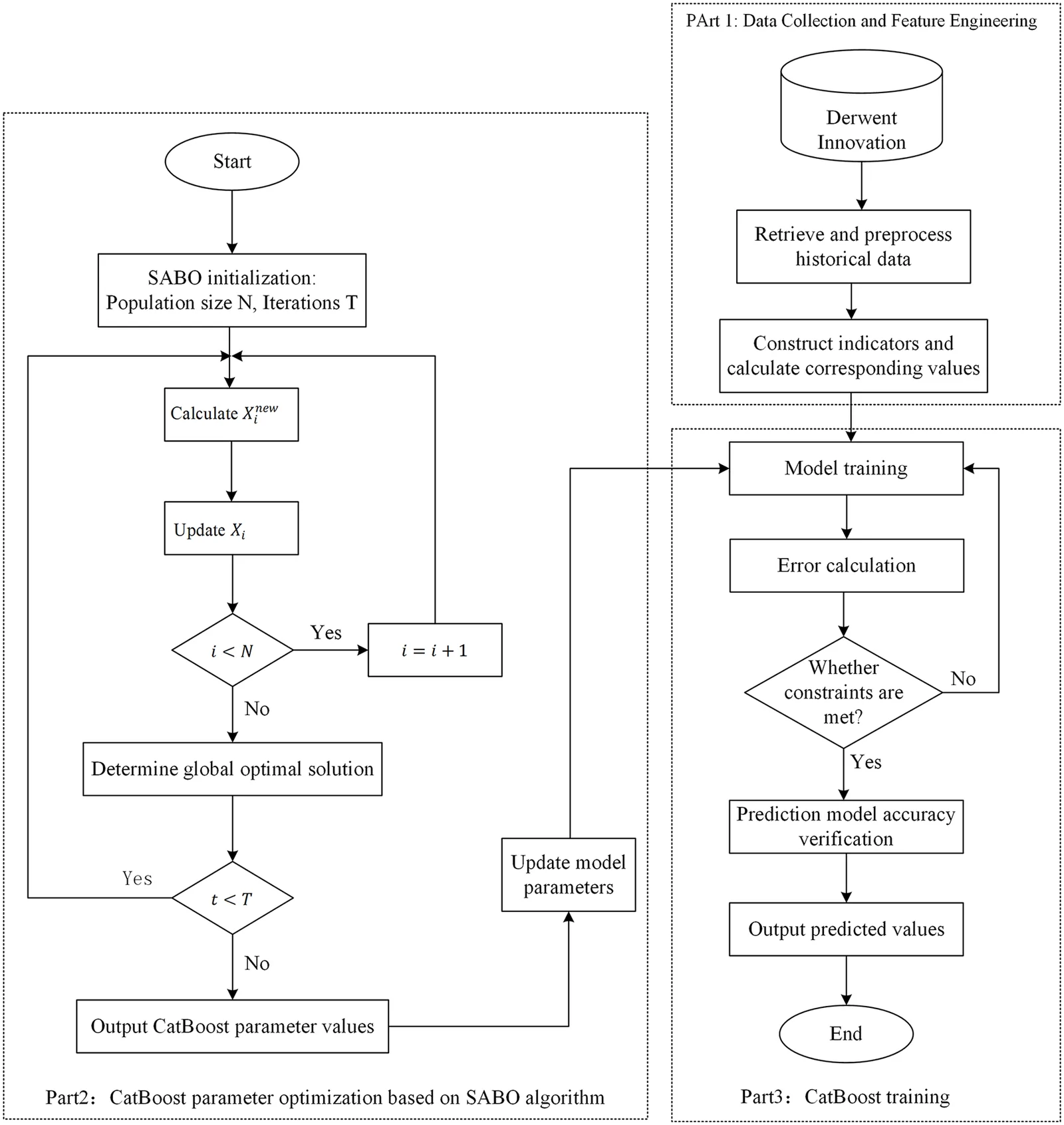
Framework of the SABO-CatBoost algorithm.

This study presents a systematic framework for the early identification of breakthrough technologies, structured around three main components. First, data are collected from Derwent Innovation and processed through feature engineering, where historical patent data are retrieved, preprocessed, and converted into structured indicators. Second, the SABO algorithm is used to optimize the hyperparameters of CatBoost by iteratively updating candidate solutions until reaching the global optimum or meeting performance constraints. Third, the optimized model is trained and evaluated on the engineered features, and SHAP analysis is applied to interpret its predictions, which reveals feature contributions and supports actionable insights. Fig 1 illustrates the overall framework.

### Part 1: Data collection and feature engineering

This section has two main objectives: first, to provide a clear and operational definition of “breakthrough technology”; and second, to develop a multidimensional framework of predictive indicators for its early identification. Historical patent data are then collected from the Derwent Innovation database, and the relevant indicators are calculated. The data are preprocessed to address missing values, outliers, and normalization as required. These features will be used to train a preliminary predictive model in the next phase.

To enable a data-driven identification model, the concept of “breakthrough technology” must first be translated into an operational and measurable definition. This study uses a classical definition for breakthrough technologies based on established patent analysis literature [26–30]. Specifically, a breakthrough technology is defined as a patent whose number of forward citations is in the top 5% of all patents filed in the same application year.

The use of forward citations as a proxy for technological significance is well established. Each forward citation indicates a knowledge flow from the cited patent to later inventions, reflecting the foundational contribution of the technology [31]. Previous studies have consistently validated citation-based measures as effective indicators of technological importance and innovation value [27,32–34].

This operational strategy balances methodological rigor and practical feasibility. Although no single metric fully captures the complexity of technological breakthroughs, the top 5% citation threshold offers a reliable and reproducible criterion for identifying patents with exceptional impact, consistent with approaches used in earlier research.

This study develops a multidimensional indicator system that relies solely on data available at the time of a patent’s publication to support early identification of breakthrough technologies. Drawing on existing literature, the system assesses inventions using both the content of the **patent document** and the **characteristics of the inventor team**, based on the idea that breakthrough potential results from the combination of technological novelty and human capital. All metrics are ex-ante and do not require future outcome data, enabling real-time evaluation of a patent’s transformative potential.

We begin with the intrinsic attributes of the patent, first considering its **Technology Breadth**, measured by the number of distinct IPC codes. A patent covering a wider range of technological fields is presumed to be more complex and valuable. In addition to breadth, radical innovation often depends on novel recombination. To capture this, we measure **Combination Novelty** as the proportion of a patent’s pair-wise IPC subclass combinations that are entirely new. This metric is based on the theory that invention results from new combinations of existing knowledge components [35].

The patent’s connection to the broader knowledge base is reflected in its citation patterns. We distinguish **Scientific Relevance**, measured by the number of citations to non-patent literature, from **Technique Relevance**, indicated by citations to prior patents. Scientific relevance signals a foundation in fundamental science, while technique relevance shows reliance on existing technical art. Both types of relevance have been empirically linked to a patent’s future impact [36,37]. In addition, the legal scope of the invention, as indicated by the claims, serves as a proxy for its technical detail and protective value, which correlates with its expected importance [33].

To provide a more detailed analysis, we move beyond counting claims and instead analyze the claims text of the patent. We introduce two new metrics based on the patent’s claims tree structure: **Protection Breadth** and **Protection Depth**. Protection Breadth, defined as the number of root-to-leaf paths in the claims tree, represents the number of distinct protected technical embodiments and reflects the strategic coverage of the technology landscape. Protection Depth, measured as the average number of nodes per path in the claims tree, quantifies the level of detail and refinement in each embodiment and indicates the precision of the legal boundaries.

To construct a claims tree, we parse each claim’s dependency references using regular expression patterns from the claim text. Independent claims (those with no preceding ‘according to claim’ clause) are treated as root nodes. For each dependent claim, we extract the first referenced parent claim and add a directed edge from that parent to the dependent claim. In practice, we observed that claims may occasionally appear out of order (e.g., a dependent claim referencing a parent claim that appears later in the document). To handle this without assuming a fixed order, we first parse all claims and store them in a dictionary keyed by claim number. For any dependent claim whose parent has not yet been parsed, we temporarily store the dependency and re-associate it after all claims have been processed. The resulting structure is a directed acyclic graph (DAG) with independent claims as roots and terminal dependent claims as leaves. The pseudocode for the construction procedure is provided in Algorithm 1.

**Algorithm 1: Claims Tree Construction**

**Input**: Set of claims C

**Output**: Directed acyclic graph G = (V, E)

1:  V ← ∅, E ← ∅, pending ← ∅

2:  **for** each claim c in C **do**

3:   **if** c is independent **then**

4:    V ← V ∪ {c}

5:   **else**

6:    p ← parent(c)

7:    **if** p exists in V **then**

8:     V ← V ∪ {c}

9:     E ← E ∪ {(p, c)}

10:    **else**

11:     pending ← pending ∪ {(p, c)}

12:    **end if**

13:   **end if**

14:  **end for**

15:  **for** each (p, c) in pending **do**

16:   **if** p exists in V **then**

17:    V ← V ∪ {c}

18:    E ← E ∪ {(p, c)}

19:   **else**

20:    Log warning: “Parent claim not found”

21:   **end if**

22:  **end for**

23:  **return** G

**Fig 2:**
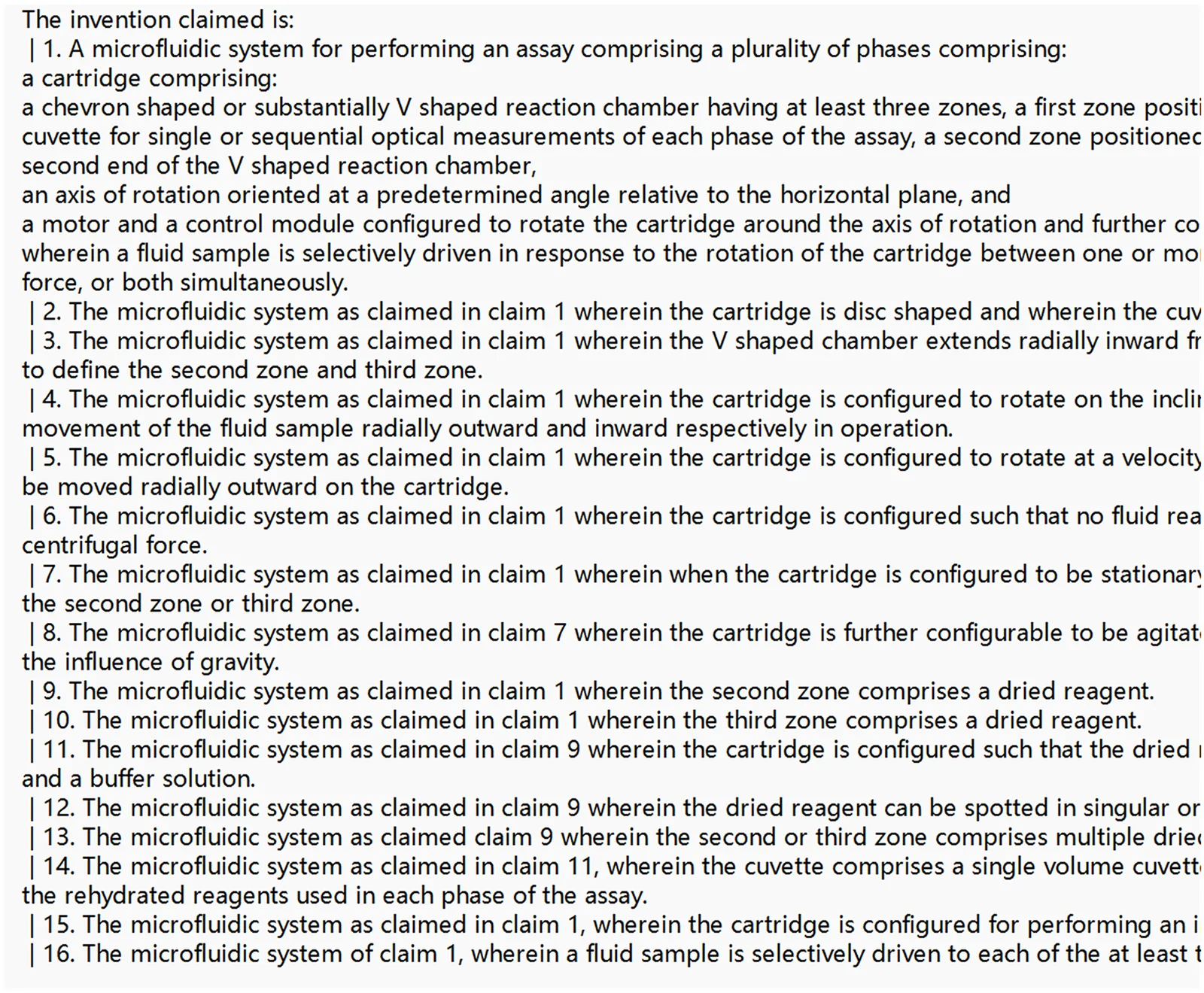
Sample patent claims text.

**Fig 3:**
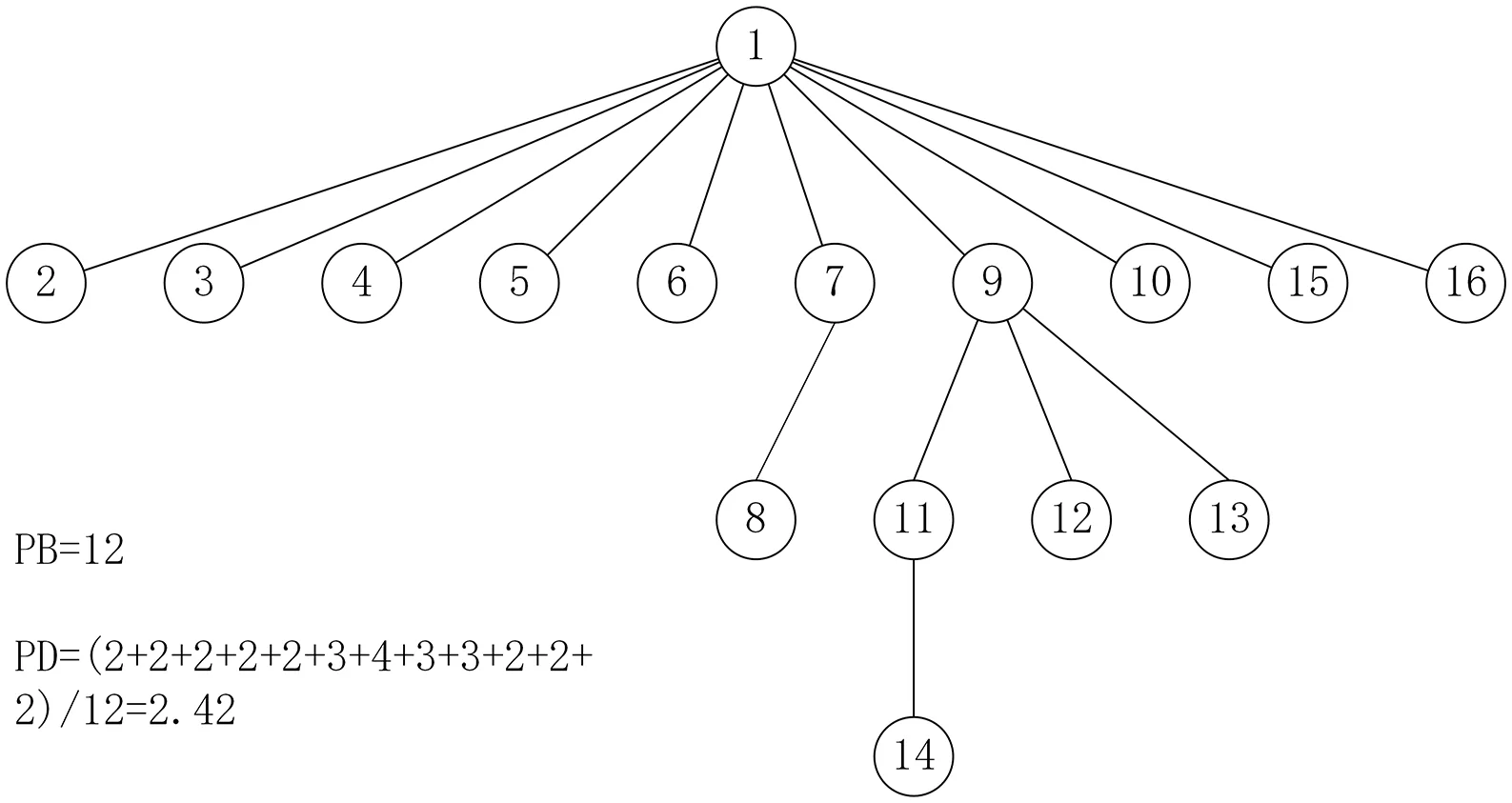
How to calculate protection breadth and protection depth.

Fig 3 illustrates the claims tree derived from Fig 2. Claim 1 is the root. Claims 2, 3, 4, 5, 6, 7, 9, 10, 15, and 16 are direct children of claim 1. Claim 8 depends on claim 7; claims 11, 12, and 13 depend on claim 9; and claim 14 depends on claim 11. The tree yields 12 root-to-leaf paths (PB = 12), with an average path length of 2.42 (PD = 2.42). (see Figs 2 and 3).

**Refinement Span** is an important temporal measure that captures the intensity and persistence of inventive activities. Defined as the period between a patent’s earliest priority date and its latest filing date, Refinement Span indicates the continuity and repeated effort invested in improving a technology. This approach is based on innovation process theory, which identifies development duration as a key factor influencing an invention’s maturity and eventual impact [38].

We analyze the structure and capabilities of the inventor team, focusing on the human agents behind the invention. **Team Size** is a key characteristic, as larger teams are theorized to bring greater diversity of ideas and problem-solving approaches [39]. The team’s experience shapes the quality of their collective effort, which we examine in terms of depth and breadth. **Average Experience**, measured as the average number of prior patents per inventor, indicates the team’s cumulative expertise in the invention process and is associated with more impactful outcomes [40]. At the same time, **Experience Diversity** (the range of technology classes in which the team has previously worked) measures their collective cognitive breadth and enables them to draw analogies from different fields [40].

Finally, the team’s **Geographic Dispersion**, measured by the Herfindahl-Hirschman index, is included. Geographically distributed teams are thought to access more diverse knowledge pools and institutional contexts, which can be an important resource for increasing the novelty and impact of their collaborative output [41,42]. Together, these indicators form a composite profile of a technology’s potential for breakthrough. Table 1 summarizes these indicators.

**Table 1:** Evaluation indicators for breakthrough patents identification.

Abbreviation	Variable	Description
TB	Technological Breadth	Number of distinct IPC codes
CN	Combination Novelty	The proportion of entirely new IPC pairs
SR	Scientific Relevance	Number of citations to non-patent literature
TR	Technique Relevance	Number of citations to patent
PB	Protection Breadth	The total number of root-to-leaf paths in the patent’s claims tree
PD	Protection Depth	The average number of nodes per root-to-leaf path in the patent’s claims tree
RS	Refinement Span	Year gap between a patent’s application filing date and its earliest priority date
TS	Team Size	Number of inventors
AE	Average Experience	Average number of prior patents by the focal patent’s inventors
ED	Experience Diversity	Number of technology classes at least one of the focal patent’s inventors has patented in before
GD	Geographic Dispersion	Herfindahl-Hirschman index of inventor geographic distribution

### Part 2: Hyperparameter optimization of the catboost model based on the SABO algorithm

This component improves model performance using intelligent optimization algorithms. The **SABO (Subtraction-Average-Based Optimizer)** is initialized by setting the population size N and the number of iterations T. The iterative optimization loop then begins: new parameter solutions are calculated, parameter values are updated, individual fitness is evaluated, and the global optimal solution is determined. The loop continues until the termination criteria are met, and the globally optimized CatBoost hyperparameter set is output. The SABO algorithm increases the model’s sensitivity and robustness in detecting early signals of breakthrough potential by efficiently exploring the hyperparameter space.

#### CatBoost as classifier.

The selection of CatBoost as the core predictive model is strategically aligned with the key requirements of our research objectives. CatBoost’s computational efficiency, driven by its native and efficient handling of categorical features without the need for extensive preprocessing (e.g., one-hot encoding) and its symmetric tree construction, enables the intensive, iterative hyperparameter optimization with SABO necessary for robust model development [12]. In terms of predictive performance, CatBoost’s “Ordered Boosting” mechanism effectively mitigates target leakage and prediction shift—a common issue in standard gradient boosting—ensuring superior generalization. This framework expertly captures the complex, nonlinear interactions within our structured bibliometric feature space while its robust regularization reduces the risk of overfitting, supporting accurate signal detection. Furthermore, CatBoost provides inherent support for multiple feature importance measures, which seamlessly underpins our use of SHAP analysis to interpret the principal factors influencing breakthrough predictions beyond mere classification [43].

Collectively, these attributes (**computational efficiency tailored for datasets with categorical variables, high predictive accuracy through bias-reducing Ordered Boosting,** and **intrinsic support for model interpretability**) establish CatBoost as a suitable and sophisticated methodological foundation for the early-stage identification of breakthrough technologies.

#### SABO as Optimizer with Lévy-flight (L-SABO).

For hyperparameter optimization, we use the SABO algorithm [11], which operates on a distinct population-based update principle centered on a special “v-subtraction” operator:


$$\begin{array}{c} A{-}_{v}B=\text{sign}\left\{\left[F\left(A\right)-F\left(B\right)\right]\left(A-\vec{v}*B\right)\right\}, \end{array}$$ (1)

where *v* is a 1-dimensional vector randomly generated from the set {1,2}, sign is the sign function, *F*(***A***) and *F*(***B***) are the fitness values of individuals ***A*** and ***B***, respectively, in the search space, and the operation “*” represents the Hadamard product of the two vectors.

Unlike traditional optimizers that depend mainly on the global best solution, SABO updates each search agent’s position using the arithmetic mean of its vector differences from all other agents in the population. This approach enables a more balanced exploration of the search space by distributing update influences across the entire population, rather than focusing on a single elite agent. As a result, SABO reduces the risk of premature convergence, which is especially important when tuning the complex hyperparameter landscape of CatBoost for our prediction task. This is formalized as follows:


$$\begin{array}{c} A^{\prime }=A+r_{i}\frac{\text{1}}{G}\sum_{j=\text{1}}^{G}\left(A{-}_{v}B\right), \end{array}$$ (2)

where $r_{i}$ is a 1-dimensional vector with random values drawn from a normal distribution in the range [0,1], and $A^{\prime }$ is the new position after the update. To enhance global exploration capability, we replace conventional random numbers with **Levy-distributed random numbers** for step size control:


$$\begin{array}{c} r_{i}\sim \text{Levy}(\beta ),\beta =\text{1}.\text{5}. \end{array}$$ (3)

This Levy-based step size adjustment enables occasional long jumps, while maintaining frequent short steps, effectively balancing exploration and exploitation during the CatBoost hyperparameter tuning process. The search is guided by a fitness function $F_{\text{fitness}}$, which is defined in the next section.

#### SABO-CatBoost Hyperparameter Optimization Process.

The SABO algorithm optimizes the hyperparameters of CatBoost through the following process:

Select an appropriate data set as input for the algorithm and perform the necessary data preprocessing. Initialize the tunable hyperparameters of the CatBoost model. Table 2 presents the hyperparameters to be optimized along with their initial values. These hyperparameters are assigned as the corresponding dimensional position vectors of each search agent, where each dimension represents a specific hyperparameter value. Set the population size N to 50, with each search agent representing a candidate solution (a combination of hyperparameters). Set the maximum number of iterations $T_{max}$ to 200.

**Table 2:** Partial optimization parameters and their adjustment ranges.

Parameter Name	Adjustment Range	Initial Value
iterations	[500, 1500]	1000
learning_rate	[0.05, 0.3]	0.1
depth	[6, 12]	10
l2_leaf_reg	[1, 5]	3
border_count	[200, 300]	254

During each iteration, the position vector of the search agent is updated according to the workflow and update formulas of the algorithm described above. **Identifying breakthrough technologies presents a highly imbalanced classification problem, with the minority class (breakthroughs) as the main focus. To ensure the optimized model detects these rare cases effectively, the fitness function in the SABO algorithm is designed to maximize the F1-score of the minority class directly**. The F1-score, calculated as the harmonic mean of precision and recall, balances the risk of missing true breakthroughs (low recall) and producing too many false alarms (low precision). This metric is well suited for situations where both detection sensitivity and prediction reliability are important.

Accordingly, the fitness of a hyperparameter combination *X* is defined as


$$\begin{array}{c} F_{\text{fitness}}(X)=\text{1}-{F\text{1}}_{\text{minority}}(X). \end{array}$$ (4)

To calculate the fitness value of each search agent, stratified five-fold cross-validation is performed using the model with the current hyperparameter settings. The optimal search agent and its corresponding fitness value are then output to evaluate model performance. The best model hyperparameter settings identified are used to initialize the CatBoost model for the next iteration. Through continuous iterative updates, the search agents obtain improved hyperparameter settings.

When the maximum number of iterations is reached, the algorithm terminates. The combination of hyperparameters corresponding to the global optimal solution is selected from all iterations using the fitness function as the evaluation metric. This process determines the optimal hyperparameter combination for the CatBoost model.

### Part 3: CatBoost Model Training, Validation, and Prediction

Once the optimized CatBoost model meets the performance constraints, it is validated using a hold-out test set to confirm reliability and generalization. The validated model is then deployed to predict new technological data, identifying areas with high breakthrough potential. To improve interpretability and support decision-making, explainability techniques such as **SHAP (SHapley Additive exPlanations)** are used to analyze feature importance and contribution patterns. The results are presented through analytical dashboards and summary reports, offering clear and actionable insights for technology forecasting and strategic planning.

#### SHAP additive explanations.

Although the CatBoost model provides strong predictive power, its algorithmic complexity often makes it a “black box” with limited interpretability. To address this, the SHAP algorithm is used to explain the model’s predictions and analyze the importance of each feature’s contribution to the results [43].

SHAP is an algorithm that interprets the output of ML models. Drawing on the Shapley value from cooperative game theory, it quantifies the contribution of different influencing factors to technology breakthrough prediction results. SHAP offers both local and global interpretations to explain the decision-making process of the CatBoost model, making it an effective tool for interpreting ML models. The calculation formula for SHAP values is as follows:


$$\begin{array}{c} \text{SHAP}\left(x_{i}\right)=\sum_{S\subseteq N\backslash \{i\}}\frac{|S|!\left(|N|-|S|-\text{1}\right)!}{|N|!}\left[f(S\cup \{i\})-f(S)\right], \end{array}$$ (5)

where:

$\text{SHAP}\left(x_{i}\right)$is the Shapley value of the influencing factors for technology breakthrough;

N is the set of all input features;

S is any subset of features that excludes the feature i, i.e., S⊆N\{i};

f(S) is the model’s predicted contribution of subset S;

f(S∪{i}) is the model’s predicted contribution after factor $x_{i}$ is added to subset S.

#### Evaluation Metrics.

This experiment adopts fundamental ML evaluation metrics including Precision, F1 Score, FPR, and FNR. The calculation formulae are as follows:


$$\begin{array}{c} Accuracy=\frac{TP+TN}{TP+FP+TN+FN} \end{array}$$ (6)

$$\begin{array}{c} Precision=\frac{TP}{TP+FP} \end{array}$$ (7)

$$\begin{array}{c} Recall=\frac{TP}{TP+FN} \end{array}$$ (8)

$$\begin{array}{c} F\text{1}=\frac{\text{2}\times Precision\times Recall}{Precision+Recall}. \end{array}$$ (9)

In the formulae, *TN* represents the number of negative samples correctly classified; *FP* denotes the number of positive samples misclassified; FN indicates the number of negative samples misclassified; and TP signifies the number of positive samples correctly classified. The F1 score is the harmonic mean of precision and recall.

### Case study

Since its emergence in the 1990s, biochip technology has been widely recognized as a transformative tool for genomics and precision medicine, due to its high-throughput, miniaturized, and parallel analysis capabilities. The field has experienced rapid technological change, significant innovation, and strong convergence between interdisciplinary research and commercial application. This combination of steady development and both incremental and radical innovations makes biochip technology a suitable empirical domain for identifying technological breakthroughs.

This study used the Derwent Innovation platform to collect patent data in the field of biochips. A dual screening mechanism combined keyword searches, which followed principles of comprehensiveness and precision and covered four major categories (fundamental technologies, application fields, sample/biomarker types, and cutting-edge technologies), with International Patent Classification codes to create a precise data collection system. Table 3 presents the search strategy.

**Table 3:** Search strategy.

Search Strategy
(IC=(C12Q* OR C12M* OR C07K* OR C12N* OR G01N* OR B01L* OR B81B* OR B81C* OR A61B* OR A61F*)) AND (CTB=(biochip* OR bio-chip* OR BioMEMS OR Bio-MEMS OR microfluid* OR micro-fluid* OR ((DNA OR gene OR RNA OR protein OR glycoprotein OR phosphoprotein OR antibody OR peptide OR genomic OR proteomic OR biosensor OR bio-sensor OR bioreactor OR bio-reactor OR NGS OR polynucleotide OR “biological probe” OR diagnostic OR cell OR tissue OR PCR OR qPCR OR sequencing OR “capillary electrophoresis” OR CE OR immunoassay OR immune OR implant OR biological OR biochemical OR bio-chemical OR dPCR OR point-of-care OR POC OR bacterial OR virus OR SPR OR SNP OR “single nucleotide polymorphism” OR liquid OR liquid-phase OR suspension OR bead OR screening OR profiling OR laboratory OR “surface plasmon resonance” OR CRISPR OR metabolomic OR nucleotide OR cancer OR tumor OR tumour OR clinical OR metabolism OR organ OR biomedical OR bio-medical OR vascular OR BBB OR organoid OR neural OR retina OR skin OR bone OR wound OR methylation OR oligonucleotide OR hybridization OR “nucleic acid”) W/0 (chip* OR microchip* OR micro-chip* OR array* OR microarray* OR micro-array*)) OR ((lab-on OR organ-on OR lung-on OR liver-on OR heart-on OR kidney-on OR brain-on OR tumor-on OR cancer-on OR human-on OR body-on OR gut-on OR placenta-on) W/1 chip*) OR (“micro total analysis system” OR μTAS OR micro-TAS OR MPS OR “blood-brain barrier” OR “microphysiological system” OR xMAP OR MASA OR liquichip OR electrowetting)))

All retrieved patents underwent rigorous cleaning and standardization, including deduplication, patent family consolidation, and relevance verification. The final curated data set of 12,902 patents provides a strong foundation for identifying and analyzing breakthrough technologies in the biochip domain, ensuring comprehensive coverage of both established and emerging technological trajectories.

**Fig 4:**
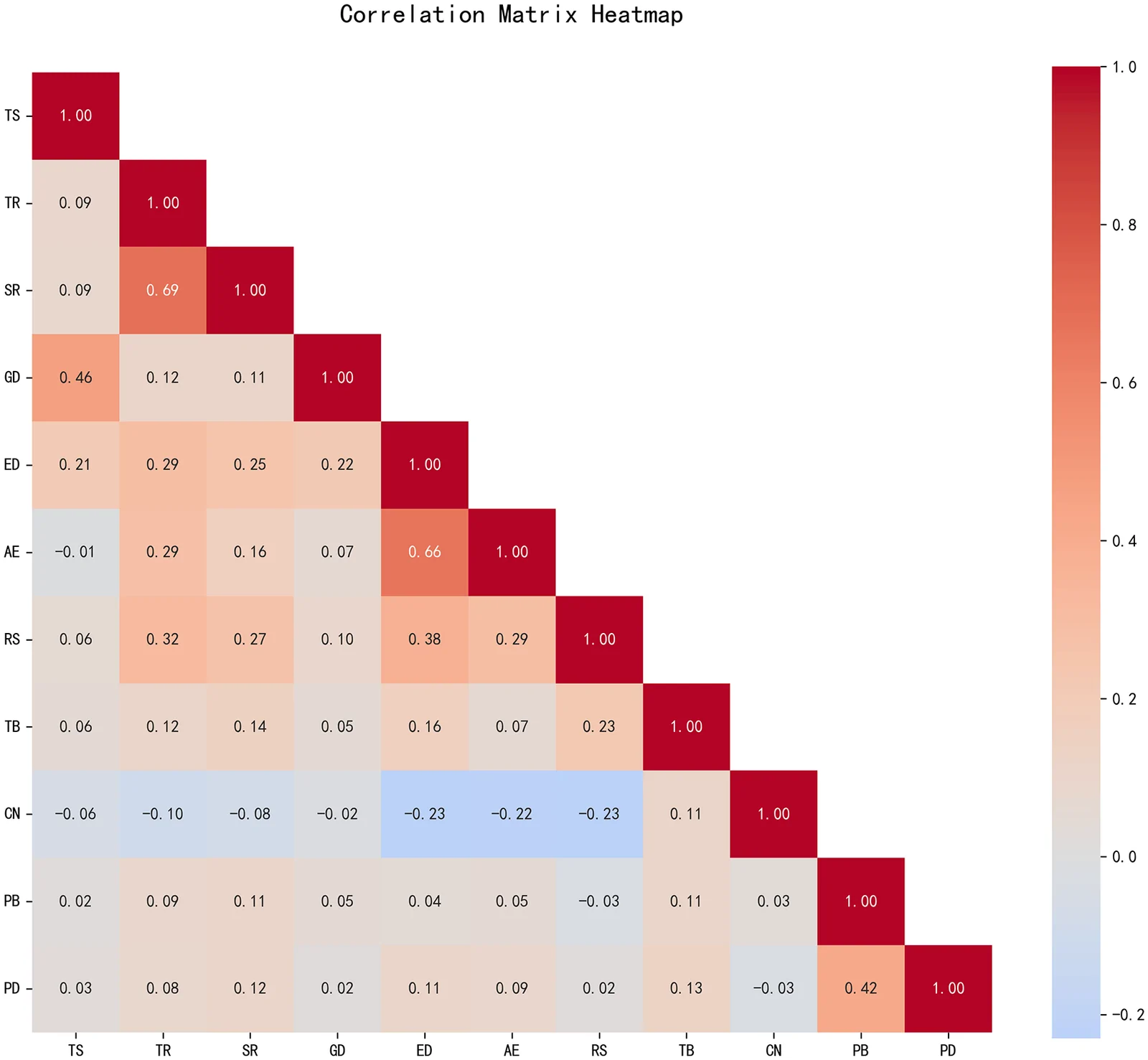
Pearson correlations matrix of variables.

After computing the indicator data, we performed a correlation analysis, as shown in Fig 4. It is generally accepted that when the absolute value of the correlation coefficient (|r| ≥ 0.8), the two factors are highly correlated and require further treatment. The results show that none of the indicator correlations exceed 0.8. Therefore, all indicators are retained, and the final indicator system includes 11 indicators.

## Implementation and result

The dataset was divided into training, validation, and test sets using a 6:2:2 ratio to support robust model evaluation. Because breakthrough patents account for only a small fraction of the dataset, we applied **SVM-SMOTE** to address the class imbalance. Unlike standard SMOTE, which generates synthetic samples uniformly along the line segment between a sample and its nearest neighbors, SVM-SMOTE leverages support vectors identified by an SVM classifier to generate synthetic samples near the decision boundary. We used the implementation from the imbalanced-learn library with k_neighbors = 5, with a sampling strategy set to target_ratio = min(0.2, neg_ratio * 1.5), where neg_ratio is the proportion of negative (majority) samples.

To assess the performance of CatBoost, we conducted comparative experiments with five other classifiers: XGBoost, LightGBM, Random Forest, SVM, and MLP. To ensure a fair comparison of out-of-the-box performance, all models were configured with their library-default hyperparameters. The sole adjustment across all models was enabling built-in class weighting (set to “balanced”) to address class imbalance. The specific default values for each model are as follows: XGBoost (n_estimators = 100, max_depth = 6, learning_rate = 0.3), LightGBM (n_estimators = 100, num_leaves = 31, learning_rate = 0.1), Random Forest (n_estimators = 100, max_depth = None), SVM (C = 1.0, kernel = ’rbf’), and MLP (hidden_layer_sizes=(100,), activation = ’relu,’ max_iter = 200).

The primary objective of this study is to accurately identify breakthrough technologies, which represent the minority (positive) class in the dataset. Therefore, the F1 score, as the harmonic mean of precision and recall, is the main metric for evaluating model performance because it balances the need to identify true breakthroughs (recall) and to minimize false alarms (precision).

As shown in Table 4, the CatBoost model achieves the highest F1 score (0.6588), indicating the best overall performance in identifying breakthrough technologies. It provides the most balanced results between precision (PRS: 0.6829) and recall (REC: 0.6364) among all models evaluated. This result demonstrates that CatBoost employs an effective classification strategy that delivers both reliable predictions and broad coverage—essential for practical technology monitoring, where both missed opportunities and false alarms can incur significant costs.

**Table 4:** Performance comparison of different models for breakthrough technology identification.

Model	ACC	PRS	REC	F1
XGBoost	0.9636	0.6532	0.6136	0.6328
LGBM	0.9624	0.6296	**0.6439**	0.6367
CatBoost	0.9663	0.6829	0.6364	**0.6588**
Random Forest	**0.9678**	**0.7882**	0.5076	0.6175
SVM	0.9403	0.4368	0.5758	0.4967
MLP	0.9601	0.6436	0.4924	0.5579

Among gradient boosting implementations, XGBoost achieved an F1 score of 0.6328, which is notably lower than that of CatBoost, indicating a measurable performance gap despite their similar algorithmic foundations. LightGBM showed comparable results, with a slightly lower F1 score of 0.6367. This ranking suggests that although all three boosting algorithms performed well relative to other methods, CatBoost’s implementation provided a modest yet meaningful advantage in handling class imbalance and capturing the complex patterns present in breakthrough technology data.

The performance of other models highlights the unique value of the boosting approach. Random Forest achieves high precision (0.7882) but detects only a limited portion of breakthroughs (REC: 0.5076), reflecting a conservative strategy unsuitable for comprehensive scanning. In contrast, MLP attains higher recall (0.4924) but much lower precision (0.6436), a trade-off that would generate excessive false leads in practice. SVM performs the worst (F1: 0.4967), underscoring its limitations in managing severe class imbalance without specific adjustments.

In conclusion, CatBoost offers the most operationally viable balance for breakthrough technology identification, effectively mitigating both oversight risk and alert fatigue. Its leading performance among gradient boosting models supports its selection as the preferred method.

### Optimzers Performance Test

**Fig 5:**
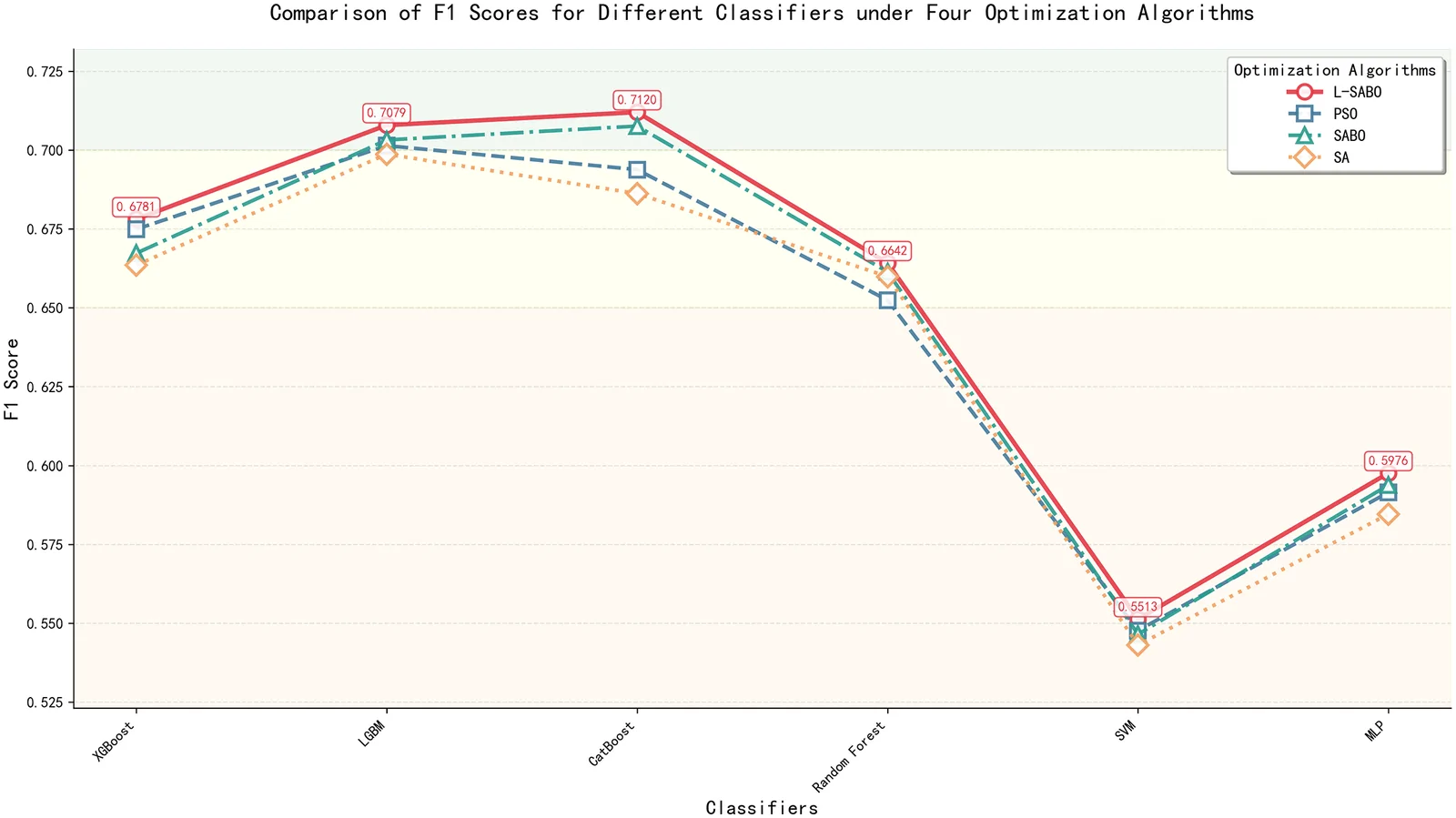
Comparison of optimal F1 scores for different classifiers under various optimization algorithms.

To assess the effectiveness of the proposed algorithm(L-SABO), we conducted comparative optimization experiments using Particle Swarm Optimization (PSO), Simulated Annealing (SA), and standard SABO across various classifiers. The F1 score for the positive class served as the primary performance metric. For each algorithm, the initial population size was set to 50, and the maximum number of iterations was set to 200 (see Fig 5).

The comparative analysis shows a clear performance ranking for all tested configurations. The CatBoost optimized with L-SABO achieved the highest F1-score at 0.7120, which is an improvement over the baseline CatBoost F1-score of 0.6588. This score is also higher than those from other optimizer-classifier combinations, including both alternative optimization methods with CatBoost and other classifiers with the L-SABO algorithm. These results indicate that the L-SABO algorithm is highly effective at searching the high-dimensional hyperparameter space of the CatBoost model, identifying a configuration that improves the balance between precision and recall for detecting breakthrough technologies. Consequently, the L-SABO-CatBoost algorithm is firmly established as the optimal model for this task.

### Visualizations of feature contribution

**Fig 6:**
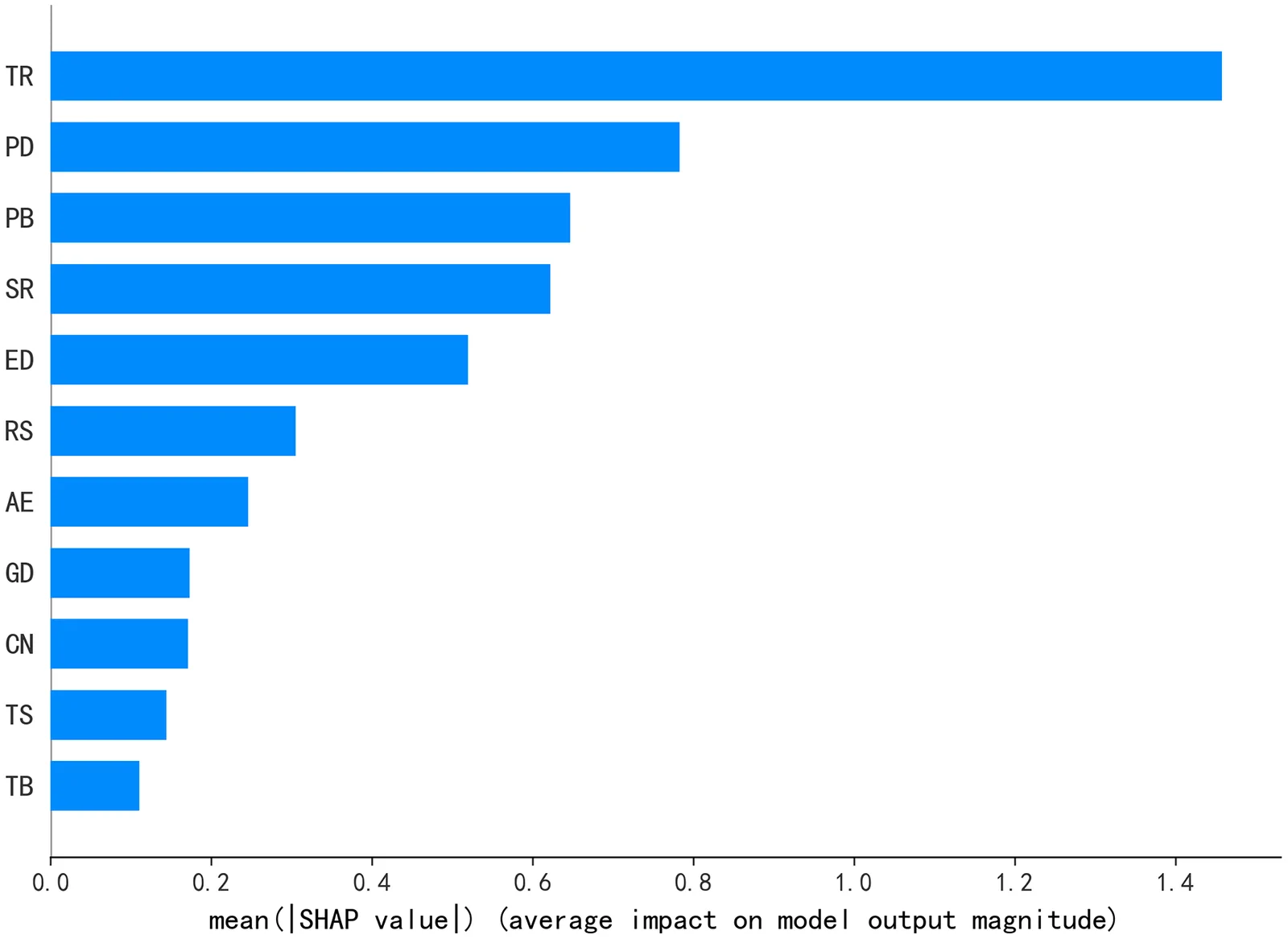
The average SHAP value of features.

**Fig 7:**
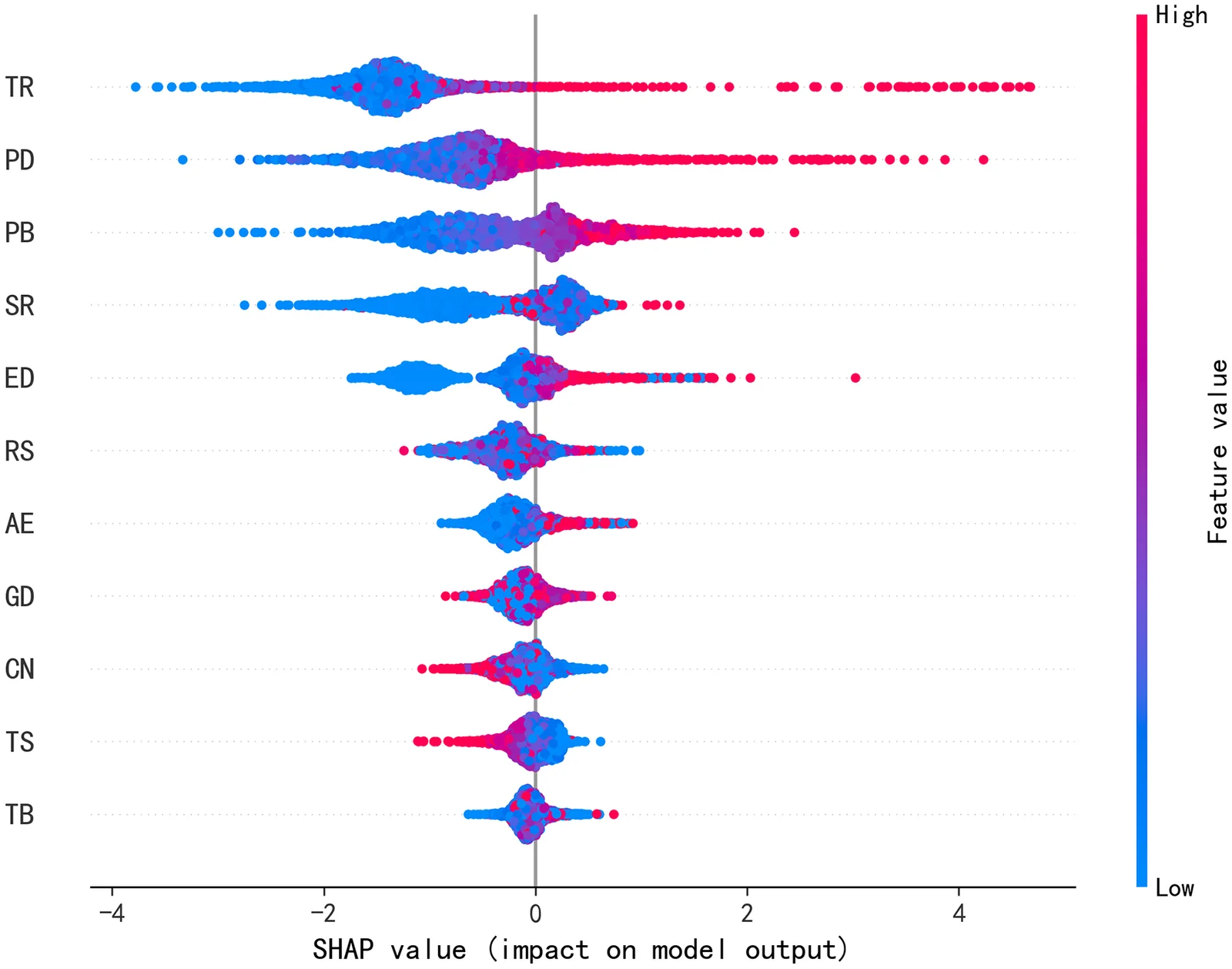
Individual feature contribution to breakthrough potential.

This study uses the SHAP method to analyze the interpretability of the breakthrough technology identification model. The global feature importance ranking, shown by the mean SHAP values in Fig 6, is: Technological Relevance (TR), Protection Depth (PD), Scientific Relevance (SR), Protection Breadth (PB), Experience Diversity (ED), Average Inventor Experience (AE), Refinement Span (RS), Geographical Dispersion (GD), Technological Breadth (TB), Combination Novelty (CN), and Team Size (TS). Fig 7 shows the distribution of individual SHAP values, which reveals the direction and heterogeneity of each feature’s impact on model predictions. These results provide a layered interpretation that aligns with existing innovation theories.

The dual SHAP analysis shows that breakthrough technologies depend on two key knowledge foundations at the same time. Technological Relevance (TR) is the most important feature, supporting the technology evolution theory proposed by Nelson and Winter [44]. TR has a strong average impact and a consistent positive effect across observations, indicating that greater engagement with prior patent literature increases the likelihood of identifying breakthroughs. Scientific Relevance (SR) is the fourth most influential feature, supporting the “Science-Technology Linkage” hypothesis proposed by Narin, et al. [45].

A key finding is the strong combined importance of patent protection characteristics. Both Protection Breadth (PB, ranked second) and Protection Depth (PD, ranked third) show significant and consistently positive influences (Fig 6). This result aligns with recent findings by Kuhn et al. [46], and supports the view that comprehensive patent claims, which include both broad coverage (multiple embodiments or applications) and detailed specification (implementation details), are reliable indicators of a technology’s foundational nature, defensive strength, and commercial potential.

Combination Novelty (CN) ranks ninth in overall feature importance, indicating that its average contribution to model predictions is modest. However, CN displays a bimodal distribution, which suggests a complex relationship with breakthrough outcomes. This pattern reflects the “double-edged sword” effect of combination novelty described by Kaplan and Vakili [28]. Their research found that moderately novel combinations tend to have the highest impact, while extremely novel combinations often face implementation challenges and lower immediate value. This finding explains the negative SHAP values observed at high CN levels.

While Mansfield identified development time as a generally positive indicator of innovation substance and maturity, our findings show an opposite relationship in the biochip domain [47]. The SHAP analysis shows that Refinement Span (RS) has a low feature importance ranking (10th), and the observed association with breakthrough potential is not consistently positive. This pattern suggests that in the rapidly evolving biochip field, extended development cycles may indicate technical obstacles, integration challenges, or misalignment with technological trajectories.

Experience Diversity (ED) and Average Inventor Experience (AE) rank fifth and sixth in overall feature importance, respectively. Both features demonstrate clear positive associations with breakthrough potential, reinforcing that teams with broader technological scope and deeper individual patenting experience are more likely to produce breakthroughs in biochips.

Geographical Dispersion (GD) ranks eighth in overall feature importance and shows a positive association with breakthrough potential. This supports the view of global innovation networks and is consistent with Kerr’s finding on the role of diaspora-driven knowledge flows, suggesting that biochip breakthroughs benefit from the integration of geographically dispersed expertise [48].

Team Size (TS) ranks seventh in overall feature importance and shows a negative association with breakthrough potential in the biochip domain. This finding contrasts with the conventional view that larger teams inherently foster innovation through greater resource and knowledge pooling. Instead, it aligns with recent large-scale evidence that the cognitive and coordination costs of managing larger groups can hinder the agility and deep focus required for breakthrough problem-solving [17]. For a complex, fast-evolving field like biochips, the results suggest that moderately sized or smaller teams may be more conducive to generating high-impact, disruptive outcomes.

## Conclusion and future research

This study presents an optimization framework developed to address the dual challenges of early breakthrough identification: detecting subtle and sparse technological signals within highly imbalanced patent data. In constructing the indicator system, we extend beyond conventional patent metrics by introducing two new indicators, Protection Breadth and Protection Depth, which are derived from the tree structure of patent claims. These indicators offer a more detailed quantification of a technology’s strategic coverage and legal precision. By integrating these features and applying the SABO metaheuristic, enhanced with Lévy-distributed step size control to improve global exploration, we directly optimize CatBoost for the minority-class F1 score. This method significantly outperforms baseline and comparator models and maintains interpretability through SHAP analysis. This work establishes a robust and scalable computational foundation for early-stage technology forecasting.

However, this study is limited to validation within a single technological domain, so further research is needed to assess the model’s generalizability across different technology domains and time periods. In addition, the model’s effectiveness depends on the availability of high-quality structured patent data. Differences in data schemas across patent databases may hinder the extraction of the proposed indicators, which presents a significant challenge for deployment.

This work’s methodological contribution is at the model level; however, fully realizing the framework’s potential requires further advances in related areas. First, recent progress in text-based and multimodal approaches for breakthrough identification highlights the importance of integrating advanced natural language processing techniques with structured multimodal data fusion, enabling the model to identify nuanced linguistic patterns and intermodal relationships in technical documents and patent drawings [10]. Second, explicit multi-order feature engineering—drawing on methods from incipient fault detection—could capture higher-order interactions among patent indicators that implicit nonlinear modeling may miss [49], while a coarse-to-fine two-stage approach could improve computational efficiency [50]. Third, adaptive mechanisms such as dynamic Lévy-flight step size adjustment and F1-score-based early stopping criteria could further refine the optimization process [51,52]. These enhancements represent promising avenues for future research.

## References

[1] Rotolo D, Hicks D, Martin BR. What is an emerging technology? Res Policy. 2015;44(10):1827–43. doi: 10.1016/j.respol.2015.06.006

[2] Fleming L. Recombinant uncertainty in technological search. Manage Sci. 2001;47(1):117–32. doi: 10.1287/mnsc.47.1.117.10671

[3] Schneider JW, Costas R. Identifying potential ‘breakthrough’ publications using refined citation analyses: Three related explorative approaches. J Assoc Inf Sci Technol. 2017;68(3):709–23. doi: 10.1002/asi.23695

[4] Kostoff RN. The use and misuse of citation analysis in research evaluation. Scientometrics. 1998;43(1):27–43. doi: 10.1007/bf02458392

[5] Schoenmakers W, Duysters G. The technological origins of radical inventions. Res Policy. 2010;39(8):1051–9. doi: 10.1016/j.respol.2010.05.013

[6] Kuhn TS. The structure of scientific revolutions. Chicago: University of Chicago Press; 1962.

[7] Min C, Bu Y, Sun J. Predicting scientific breakthroughs based on knowledge structure variations. Technol Forecast Soc Change. 2021;164:120502. doi: 10.1016/j.techfore.2020.120502

[8] Dahlin KB, Taylor M. Opportunities and challenges: a study of innovation through the lens of diversity. Manage Sci. 2005;48(2):451–70. doi: 10.1287/mnsc.1040.0357

[9] Lee C, Kwon O, Kim M, Kwon D. Early identification of emerging technologies: A machine learning approach using multiple patent indicators. Technol Forecast Soc Change. 2018;127:291–303. doi: 10.1016/j.techfore.2017.10.002

[10] Park I, Sohn SY. A novel deep learning model for predicting the value of patents. Scientometrics. 2020;125(1):829–30. doi: 10.1007/s11192-020-03653-9

[11] Trojovský P, Dehghani M. Subtraction-Average-Based Optimizer: A New Swarm-Inspired Metaheuristic Algorithm for Solving Optimization Problems. Biomimetics (Basel). 2023;8(2):149. doi: 10.3390/biomimetics8020149 37092401 PMC10123613

[12] Prokhorenkova L, Gusev G, Vorobev A, Dorogush AV, Gulin A. CatBoost: unbiased boosting with categorical features. In: Advances in Neural Information Processing Systems 31. Red Hook: Curran Associates Inc.; 2018. p. 6638–48.

[13] Häyrynen M. Breakthrough research: Funding for high-risk research at the Academy of Finland (No. 10/07). Helsinki: Publications of Academy of Finland; 2007.

[14] Winnink JJ, Tijssen RJW, van Raan AFJ. Searching for new breakthroughs in science: How effective are computerised detection algorithms? Technol Forecast Soc Change. 2019;146:673–86. doi: 10.1016/j.techfore.2018.05.018

[15] Garfield E, Welljams-Dorof A. Citation data: their use as quantitative indicators for science and technology evaluation and policy-making. Sci Public Policy. 1992;19(5):321–7. doi: 10.1093/spp/19.5.321

[16] Funk RJ, Owen-Smith J. A dynamic network measure of technological change. Manage Sci. 2017;63(3):791–817. doi: 10.1287/mnsc.2015.2366

[17] Wu L, Wang D, Evans JA. Large teams develop and small teams disrupt science and technology. Nature. 2019;566(7744):378–82. doi: 10.1038/s41586-019-0941-9 30760923

[18] Jordan MI, Mitchell TM. Machine learning: Trends, perspectives, and prospects. Science. 2015;349(6245):255–60. doi: 10.1126/science.aaa8415 26185243

[19] Yan R, Tang J, Liu X, Shan D, Li X. Citation count prediction: Learning to estimate future citations for literature. In: Proceedings of the 21st ACM International Conference on Information and Knowledge Management; 2012 Oct 29-Nov 2; Maui, Hawaii, USA. New York: ACM; 2012. p. 1247–52.

[20] Singh M, Jaiswal A, Shree P, Pal A. Citation count prediction using representation learning. In: Proceedings of the 13th International Conference on Natural Language Processing; 2015 Dec; Pune, India. 2015. p. 228–33.

[21] Chen J, Zhang C. Predicting citation counts of papers. In: 2015 IEEE 14th International Conference on Cognitive Informatics & Cognitive Computing (ICCI*CC); 2015 Jul 6-8; Beijing, China. Piscataway: IEEE; 2015. p. 434–40.

[22] Abrishami A, Aliakbary S. Predicting citation counts based on deep neural network learning techniques. J Informetr. 2019;13(2):485–99. doi: 10.1016/j.joi.2019.02.011

[23] Wang X, Yang X, Du J, Wang X, Li J, Tang X. A deep learning approach for identifying biomedical breakthrough discoveries using context analysis. Scientometrics. 2021;126(7):5531–49. doi: 10.1007/s11192-021-04003-z

[24] Li X, Wen Y, Jiang J, Daim T, Huang L. Identifying potential breakthrough research: A machine learning method using scientific papers and Twitter data. Technol Forecast Soc Change. 2022;184:122042. doi: 10.1016/j.techfore.2022.122042

[25] Wan A, Zhang F, Al-Bukhaiti K, Cheng X, Ji X, Wang J, et al. A Novel GA-PSO-SVM Model for Compound Fault Diagnosis in Gearboxes With Limited Data. IEEE Sensors J. 2025;25(16):30431–43. doi: 10.1109/jsen.2025.3576761

[26] Trajtenberg M. A Penny for Your Quotes: Patent Citations and the Value of Innovations. RAND J Econ. 1990;21(1):172. doi: 10.2307/2555502

[27] Hall BH, Jaffe AB, Trajtenberg M. Market value and patent citations: A first look. Rand J Econ. 2005;36(1):16–38. doi: 10.2139/ssrn.443040

[28] Kaplan S, Vakili K. The double-edged sword of recombination in breakthrough innovation. Strat Manag J. 2015;36(10):1435–57. doi: 10.1002/smj.2294

[29] Kelly B, Papanikolaou D, Seru A, Taddy M. Measuring Technological Innovation over the Long Run. Am Econ Rev Insights. 2021;3(3):303–20. doi: 10.1257/aeri.20190499

[30] Vestal A, Danneels E. Tracing the emergence of breakthrough inventions: A dynamic recombination approach. Res Policy. 2024;53(1):104915. doi: 10.1016/j.respol.2023.104915

[31] Jaffe AB, Trajtenberg M. Patents, citations, and innovations: A window on the knowledge economy. Cambridge: MIT Press; 2002.

[32] Ernst H. Patent information for strategic technology management. World Patent Inf. 2003;25(3):233–42. doi: 10.1016/s0172-2190(03)00077-2

[33] Lanjouw JO, Schankerman M. Patent quality and research productivity: Measuring innovation with multiple indicators. Econ J. 2004;114(495):441–65. doi: 10.1111/j.1468-0297.2004.00216.x

[34] Kogan L, Papanikolaou D, Seru A, Stoffman N. Technological Innovation, Resource Allocation, and Growth*. Q J Econ. 2017;132(2):665–712. doi: 10.1093/qje/qjw040

[35] Arthur WB. The nature of technology: What it is and how it evolves. New York: Free Press; 2009.

[36] Podolny JM, Stuart TE. A role-based ecology of technological change. Am J Sociol. 1995;100(5):1224–60. doi: 10.1086/230637

[37] Gittelman M, Kogut B. Does good science lead to valuable knowledge? Biotechnology firms and the evolutionary logic of citation patterns. Manag Sci. 2003;49(4):366–82. doi: 10.1287/mnsc.49.4.366.14420

[38] Van de Ven AH, Angle HL, Poole MS. Research on the management of innovation: The Minnesota studies. New York: Oxford University Press; 1999.

[39] Taylor A, Greve HR. Superman or the fantastic four? Knowledge combination and experience in innovative teams. Acad Manag J. 2006;49(4):723–40. doi: 10.5465/amj.2006.22083029

[40] Singh J, Fleming L. Lone inventors as sources of breakthroughs: Myth or reality? Manage Sci. 2010;56(1):41–56. doi: 10.1287/mnsc.1090.1072

[41] Lahiri N. Geographic distribution of R&D activity: How does it affect innovation quality? Acad Manag J. 2010;53(5):1194–209. doi: 10.5465/amj.2010.54533233

[42] Tzabbar D, Vestal A. Bridging the social chasm in geographically distributed R&D teams: The moderating effects of relational strength and status asymmetry on the novelty of team innovation. Organ Sci. 2015;26(3):811–29. doi: 10.1287/orsc.2015.0969

[43] Lundberg SM, Lee SI. A unified approach to interpreting model predictions. In: Guyon I, Luxburg UV, Bengio S, Wallach H, Fergus R, Vishwanathan S, et al., editors. Advances in Neural Information Processing Systems 30. Red Hook: Curran Associates Inc.; 2017. p. 4765–74.

[44] Nelson RR, Winter SG. An evolutionary theory of economic change. Cambridge: Harvard University Press; 1982.

[45] Narin F, Hamilton KS, Olivastro D. The increasing linkage between U.S. technology and public science. Res Policy. 1997;26(3):317–30. doi: 10.1016/s0048-7333(97)00013-9

[46] Kuhn JM, Thompson NC. How to measure and draw causal inferences with patent scope. Int J Econ Bus. 2019;26(1):5–38. doi: 10.1080/13571516.2018.1553284

[47] Mansfield E, Rapoport J, Schnee J, Wagner S, Hamburger M. Research and innovation in the modern corporation. London: Palgrave Macmillan; 1971.

[48] Kerr WR. Breakthrough inventions and migrating clusters of innovation. J Urban Econ. 2010;67(1):46–60. doi: 10.1016/j.jue.2009.09.006

[49] Wang M, Cheng F, Xie M, Qiu G, Zhang J. Intensive Multiorder Feature Extraction for Incipient Fault Detection of Inverter System. IEEE Trans Power Electron. 2025;40(2):3543–52. doi: 10.1109/tpel.2024.3487853

[50] Qian Y, Wang Q, Yin L, Lu A-Y. CF-DTI: coarse-to-fine feature extraction for enhanced drug-target interaction prediction. Health Inf Sci Syst. 2025;13(1):55. doi: 10.1007/s13755-025-00370-6 40919585 PMC12408429

[51] Guo J, Li Y, Huang B, Ding L, Gao H, Zhong M. An online optimization escape entrapment strategy for planetary rovers based on Bayesian optimization. J Field Robot. 2024;41(8):2518–29. doi: 10.1002/rob.22361

[52] Ding X, Xu Y, Li G, Yang K, Yuan J, An J. Design and Performance Evaluation for BILCM-ID System With Improved Stopping Criterion. IEEE Trans Veh Technol. 2025;74(4):6779–84. doi: 10.1109/tvt.2024.3519540

